# Enhancing Salt Tolerance in Soybean by Exogenous Boron: Intrinsic Study of the Ascorbate-Glutathione and Glyoxalase Pathways

**DOI:** 10.3390/plants10102085

**Published:** 2021-10-01

**Authors:** Hesham F. Alharby, Kamrun Nahar, Hassan S. Al-Zahrani, Khalid Rehman Hakeem, Mirza Hasanuzzaman

**Affiliations:** 1 Department of Biological Sciences, Faculty of Science, King Abdulaziz University, Jeddah 21589, Saudi Arabia; halharby@kau.edu.sa (H.F.A.); hsalzahrani@kau.edu.sa (H.S.A.-Z.); kur.hakeem@gmail.com (K.R.H.); 2 Department of Agricultural Botany, Sher-e-Bangla Agricultural University, Dhaka 1207, Bangladesh; knahar84@yahoo.com; 3 Department of Agronomy, Faculty of Agriculture, Sher-e-Bangla Agricultural University, Dhaka 1207, Bangladesh

**Keywords:** trace element, plant nutrient, salinity, antioxidant defense system, glyoxalase system

## Abstract

Boron (B) performs physiological functions in higher plants as an essential micronutrient, but its protective role in salt stress is poorly understood. Soybean (*Glycine max* L.) is planted widely throughout the world, and salinity has adverse effects on its physiology. Here, the role of B (1 mM boric acid) in salt stress was studied by subjecting soybean plants to two levels of salt stress: mild (75 mM NaCl) and severe (150 mM NaCl). Exogenous B relieved oxidative stress by enhancing antioxidant defense system components, such as ascorbate (AsA) levels, AsA/dehydroascorbate ratios, glutathione (GSH) levels, the GSH and glutathione disulfide ratios, and ascorbate peroxidase, monodehydroascorbate reductase, and dehydroascorbate reductase activities. B also enhanced the methylglyoxal detoxification process by upregulation of the components of the glyoxalase system in salt-stressed plants. Overall, B supplementation enhanced antioxidant defense and glyoxalase system components to alleviate oxidative stress and MG toxicity induced by salt stress. B also improved the physiology of salt-affected soybean plants.

## 1. Introduction

Soybean (*Glycine max* L.) is a widely grown legume in America, Asia, Europe, and Africa and is consumed mostly as oil and soy protein [1]. However, soybean cultivation is now increasingly hampered due to various environmental stresses, including salinity, in many soybean growing areas. Climate change, anthropogenic activities, and poor agronomic management continue to increase the occurrence of salt stress in the crops growing in the field [2]. Salt-affected soils are distinguished by the presence of considerable amounts of soluble salts that are taken up by the plants growing there. This salt accumulation results in plant water stress due to disrupted osmotic potential, as well as interrupted uptake of essential nutrients and disturbance of the plant ionic balance [3]. Biochemical and physiological changes due to salt stress inhibit germination and post-germination development, with decreased root and shoot growth as common consequences of salt stress. The decreases in water absorption and mineral nutrients, the disruption of ionic and nutrient homeostasis, and the induction of ion toxicity lead directly and indirectly to growth inhibition [4]. Different physiological processes, including transpiration, photosynthesis, and translocation of assimilates, are hindered under salinity [5]. Therefore, altered developmental processes and yield reduction are key consequences of salt stress. Methylglyoxal generation can be increased 2- to 6-fold under abiotic stress [6].

Salt stress disrupts the equilibrium in osmotic potential as well as of different ions. Salt stress has injurious effects on chlorophyll (Chl) biosynthesis as well as on the efficiency of photosynthesis. The photon energy, therefore, cannot be appropriately used by photosystems, and plants suffer from oxidative stress due to excess production of reactive oxygen species (ROS) [3,7]. Ionic/nutrient imbalances alter the biosynthesis or metabolism of essential metabolites and disrupt the activity of enzymes involved in biochemical and physiological processes, further exacerbating ROS generation and oxidative damage in plants under salinity [4,8,9]. Many previous scientific papers provided evidence that oxidative stress tolerance is a prerequisite for enhancing overall stress tolerance, including salt stress [10]. 

Plants are equipped with antioxidant defense systems that protect against ROS generation, but these systems are only effective up to a certain limit. The antioxidant defense system includes both enzymatic and nonenzymatic components [10], and enhancement of this defense mechanism is a vital strategy for improving salt tolerance [10,11]. One metabolite that is involved in these defense systems is methylglyoxal (MG), a reactive substance produced as a by-product of different metabolic pathways in the chloroplast, mitochondria, and cytosol. Recently, the importance of the glyoxalase system has been prioritized for enhancing stress tolerance [8,10]. 

The effects of salt stress on mineral metabolism have received particular attention, but more study is required on micronutrients such as boron (B). Boron is a micronutrient that performs different physiological and developmental functions in plants. Boron deficiency decreases photosynthesis, pollen formation, nucleic acid biosynthesis, starch metabolism, and auxin function. Plant growth, including root elongation, is adversely affected by B deficiency. Plasmalemma-bound enzyme activities and the plasma membrane potential are altered due to B deficiency. Boron plays a role in protein configuration, the synthesis of phenolic compounds, and nitrogen metabolism [12,13,14]. A few recent research findings have demonstrated that supplementation with B can provide stress protection in plants by regulating physiological processes, improving the structural integrity of cytosolic organs, and enhancing antioxidant defense systems [3,9,15,16]. The aim of the present study was to investigate the antioxidant defense, the glyoxalase system, and the physiological attributes of salt-stressed soybean and the regulatory function of B in improving soybean performance under salt stress.

## 2. Materials and Methods

### 2.1. Growing Condition and Treatments

Soybean seeds were sown in plastic pots (63.5 cm depth and 26 cm diameter) in soil amended with organic manure and fertilizers at recommended doses [17]. After germination, five plants were allowed to grow in each pot. Two sets of pots were grown at the same time and under the same conditions. One set was used for morphological and growth studies, and the other was used for physiological and biochemical investigations. Twenty-day-old plants were treated with 75 mM NaCl (mild stress) and 150 mM NaCl (severe stress). A separate set of control plants was grown without added NaCl. The control and salt-treated seedlings were administered B (foliar application of 1 mM boric acid at one-week intervals up to 50 days after sowing [DAS]). A completely randomized design (CRD) was executed with three replications.

### 2.2. Growth Parameters 

Plant height was measured using a measuring tape, and the average from five plants was considered for each replication. 

Leaf photographs were captured with a digital camera, and Image-J software was used to calculate the leaf area [18].

Five sample plants were harvested from each pot (per replication) following completion of the treatments (45 DAS). Shoot fresh weight (FW) plant^−1^ was determined, and then the plants were oven-dried at 72 °C for 48 h for dry weight (DW) plant^−1^ determinations. 

### 2.3. Measurement of Physiological Parameters

Five leaves were randomly selected from a plant from each pot of each replication to record the Soil Plant Analysis Development (SPAD) value with a SPAD meter (atLEAF, USA). Three SPAD readings were taken at different positions on a leaf. The Chl content was then calculated according to the conversion factor suggested by the user manual and following Zhu et al. [19]. 

The relative water content (RWC) [20] was recorded from whole leaf discs, which were weighed for FW and then floated in distilled water in petri plates and kept in the dark. The leaf weight was determined again after 8 h, after blotting excess water with tissue paper, and was treated as the turgid weight (*TW*). Dry weights were determined after drying at 80 °C (for 48 h), and the RWC was calculated using the following formula

$$\mathrm{RWC}\;\left(\%\right)=\frac{FW-DW}{TW-DW}\times 100$$

Electrolyte leakage (EL) was recorded at 30 DAS by placing a 0.5 g leaf sample into a Falcon tube containing distilled water and placing the tubes in a water bath at 40 °C for 1 h. After cooling, the electrical conductivity (*EC*_1_) was recorded with a conductivity meter. The samples were heated again at 121 °C in an autoclave for about 1 h, and the electrical conductivity (*EC_2_*) was measured again after cooling the samples. The formula for computing the EL was [21]

$$\mathrm{EL}\left(\%\right)=\frac{EC1}{EC2}\times 100$$

The proline (Pro) content was estimated by homogenizing leaf tissue with sulfosalicylic acid and centrifuging (11,500× *g*, 4 °C, 15 min). An aliquot of the supernatant was mixed with acid ninhydrin and glacial acetic acid and heated at 100 °C in a water bath for 60 min. After cooling, toluene was added, the contents were vortexed, and the toluene layer containing the Pro chromophore was optically assayed at 520 nm [22]. The Pro content was calculated using a standard curve made from known Pro concentrations (10, 20, 40, 60, and 80 µg mL^−1^).

### 2.4. Estimation of Lipid Peroxidation and H_2_O_2_ Concentration

Lipid peroxidation was determined from the malondialdehyde (MDA) level. Leaves were homogenized in 5% (*w*/*v*) trichloroacetic acid (TCA) and centrifuged at 11,500× *g* for 15 min. The supernatant was mixed with thiobarbituric acid (TBA), heated at 95 °C for 60 min, cooled in an ice bath, and centrifuged again for 10 min. The absorbance of the supernatant was read at 532 nm to determine the malondialdehyde content. The measurements were corrected by deducting the absorbance at 600 nm, where the extinction coefficient was 155 mM^−1^ cm^−1^ [23].

The H_2_O_2_ was estimated by homogenizing leaves in potassium phosphate (K-P) buffer (pH 6.5) at 4 °C and centrifuging at 11,500× *g*. The supernatant was added to a mixture of TiCl_4_ and H_2_SO_4_ and let stand at room temperature. After centrifuging at 11,500× g, the optical absorption of the supernatant was recorded spectrophotometrically at 410 nm to determine the H_2_O_2_ level [24].

### 2.5. Ascorbate and Glutathione Estimation

Leaves were homogenized in 5% meta-phosphoric acid containing 1 mM EDTA and centrifuged at 11,500× *g* (15 min; 4 °C) and the supernatant was analyzed for ascorbate [25,26] and glutathione [24,25,27]. Total AsA was estimated by reducing the oxidized portion with 0.1 M dithiothreitol (1 h at room temperature), and the supernatant was read at 265 nm using 1.0 unit ascorbate oxidase (AO). The ratio of oxidized AsA/dehydroascorbate (DHA) was calculated by deducting the reduced AsA from the total AsA. A standard curve was prepared from different concentrations of AsA (400, 600, and 800, and 1000 µM) to determine the AsA content of the plant samples. 

GSH content was determined by a previously published method [24] with some modifications [27]. The supernatant was neutralized with K-P buffer (0.5 M) (pH 7). Then, after oxidizing GSH with 5,5-dithio-bis (2-nitrobenzoic acid) (DTNB) and reducing with nicotinamide adenine dinucleotide phosphate (NADPH) in the presence of glutathione reductase (GR), the supernatant was used to measure total GSH content spectrophotometrically at 412 nm. Oxidized GSH (GSSG) content was obtained after removing GSH with 2-vinylpyridine. The GSH level was obtained by subtracting GSSG from total GSH. Standard curves of known concentrations of GSH (20, 40, 60, and 80 µg mL^−1^) and GSSG (12, 16, 20, and 24 µg mL^−1^) were used to determine the contents in plant samples. 

### 2.6. Assays of Enzymes of the AsA-GSH Pathway and Glyoxalase System

The protein contents of the plant samples were determined [28] by grinding leaf tissue in 50 mM K–P (potassium phosphate) buffer (pH 7.0) containing 100 mM KCl, 1 mM AsA, 5 mM β-mercaptoethanol, and 10% (*w*/*v*) glycerol and centrifuging at 11,500 ×*g*. The supernatants were utilized for enzyme activity determinations. 

Ascorbate peroxidase (APX, EC: 1.11.1.11) activity was measured in K-P buffer (pH-7) containing H_2_O_2_, EDTA, and AsA. The absorbance was read at 290 nm, using 2.8 mM^–1^ cm^–1^ as the extinction coefficient [29].

The activity of monodehydroascorbate reductase (MDHAR, EC: 1.6.5.4) was measured in an assay buffer of Tris-HCl buffer, AsA, nicotinamide adenine dinucleotide (NADPH), and AO and recording the absorbance at 340 nm, using 6.2 mM^–1^ cm^–1^ as the extinction coefficient [25].

A previously published method [29] was used to determine dehydroascorbate reductase (DHAR, EC: 1.8.5.1) activity in an assay solution containing DHA, K-P buffer, and GSH and reading the absorbance at 265 nm using 14 mM^–1^ cm^–1^ as the extinction coefficient.

Glutathione reductase (GR, EC: 1.6.4.2) activity was measured in assay buffer containing NADPH, GSSG, K-P buffer pH 7, and EDTA and reading the absorbance at 340 nm using an extinction coefficient of 6.2 mM^–1^ cm^–1^ [25].

A previously published method [25] was used to determine the activity of glyoxalase I (Gly I, EC: 4.4.1.5) in an assay mixture containing GSH, K-P buffer, MG, and magnesium sulfate and reading the absorbance at 240 nm using 3.37 mM^–1^ cm^–1^ as the extinction coefficient. Glyoxalase II (Gly II, EC: 3.1.2.6) activity was determined using an assay buffer containing *S*-d-lactoyl glutathione, Tris-HCl buffer, and EDTA [25] and reading the absorbance at 412 nm using 13.6 mM^–1^ cm^–1^ as the extinction coefficient.

### 2.7. Methylglyoxal Level

Leaves were homogenized in 5% perchloric acid, centrifuged at 11,000× *g*, and the supernatant was decolorized and neutralized. The MG level was determined by adding sodium dihydrogen phosphate and *N*-acetyl-l-cysteine. The developed *N*-α-acetyl-*S*-(1-hydroxy-2-oxo-prop-1-yl)cysteine product was read after 10 min at 288 nm [4].

### 2.8. Statistical Analysis

Data were evaluated by analysis of variance (ANOVA) using CoStat v.6.400 software [30]. Means were separated according to Tukey’s honestly significant difference (HSD) test at *p* ≤ 0.05.

## 3. Results

### 3.1. Growth Parameters

**Figure 1:**
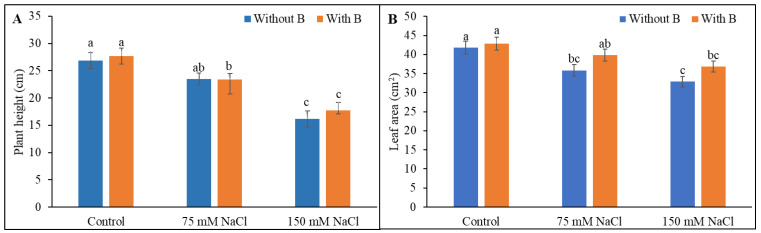
Plant height (**A**) and leaf area (**B**) of salt-stressed soybean following B supplementation. Twenty-day-old plants were subjected to two levels of salt (75 and 150 mM NaCl, 30 days) and supplemented with B (1 mM boric acid). Control treatments were grown without salt. Mean (±SD) was computed from three replicates for each treatment. Different letters over the bars indicate significant differences at *p* ≤ 0.05 applying Tukey’s HSD test.

Plant height was reduced by 13% and 40% by exposure to mild and severe salt stress, respectively, compared to unstressed control plants. Boron addition had no significant effect on the plant height of salt-treated plants (Figure 1A). Leaf area decreased at both levels of salt stress, and B addition had no significant effect on the leaf area of the salt-treated plants (Figure 1B). 

**Figure 2:**
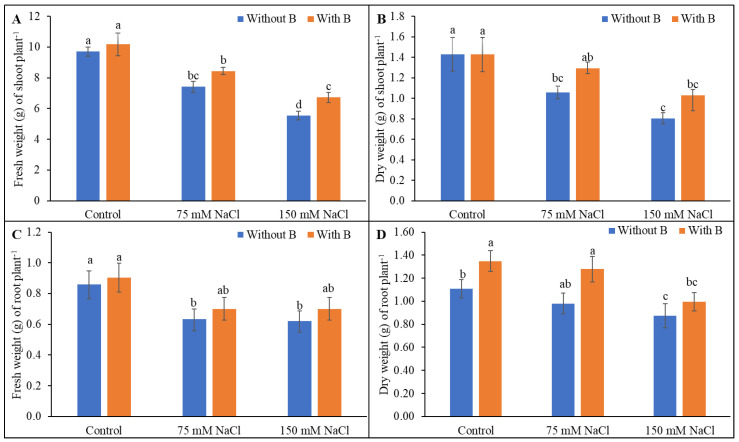
Shoot fresh weight (**A**), shoot dry weight (**B**), root fresh weight (**C**), and root dry weight (**D**) of soybean as affected by salt stress with or without B supplementation. Twenty-day-old plants were imposed with two levels of salt (75 and 150 mM NaCl, 30 days) and a set of plants were supplemented with B (1 mM boric acid). Control treatments were grown without salt. Mean (±SD) was computed from three replicates for each treatment. Different letters over the bars indicate significant differences at *p* ≤ 0.05 applying Tukey’s HSD test.

Salt stress reduced shoot and root FW and DW compared to unstressed controls treatment. The decreased shoot FW under 150 mM salt stress was restored by B supplementation (Figure 2). Boron addition did not significantly mitigate the reductions in shoot dry weight, root fresh weight, or root dry weight induced by salt stress. 

### 3.2. Physiological Parameters

**Figure 3:**
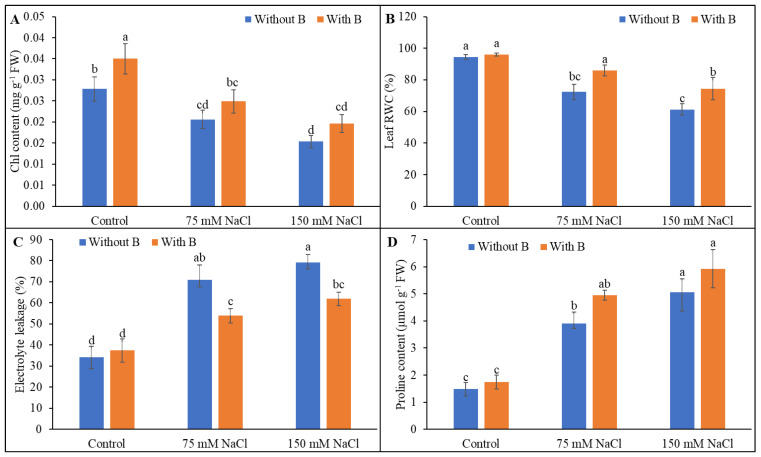
Chl content (**A**), leaf relative water content (**B**), electrolyte leakage (**C**) and proline content (**D**) of soybean as affected by salt stress with or without B supplementation. Twenty-day-old plants were imposed with two levels of salt (75 and 150 mM NaCl, 30 days) and a set of plants were supplemented with B (1 mM boric acid). Control treatments were grown without salt. Mean (±SD) was computed from three replicates for each treatment. Different letters over the bars indicate significant differences at *p* ≤ 0.05 applying Tukey’s HSD test.

Chlorophyll breakdown is common under salt stress, and the Chl content was decreased at both levels of salt stress compared with the unstressed controls. The addition of B to salt-treated seedlings increased the Chl level by 21% and 28% in the mild and severe salinity treatments, respectively, but these differences were not statistically significant compared to the salt-stressed seedlings without B supplementation (Figure 3A).

Leaf RWC was decreased by salt treatment, but the relative water content was increased by 19% and 22% by B supplementation in plants treated with mild and severe salt stress (Figure 3B). Electrolyte leakage is a recognizable indicator of oxidative damage, and soybean plants showed a drastic rise in electrolyte leakage of 107% and 131% when subjected to mild and severe salinity treatments, respectively, compared to the unstressed controls. Supplementation with B noticeably diminished electrolyte leakage by 24% and 22% in the mild and severe salinity conditions, respectively, compared to salt stress alone (Figure 3C).

The Pro content increased upon exposure to salinity, and the level was further increased by B addition, but that increase was not statistically significant (Figure 3D).

### 3.3. Malondialdehyde and H_2_O_2_ Levels

**Figure 4:**
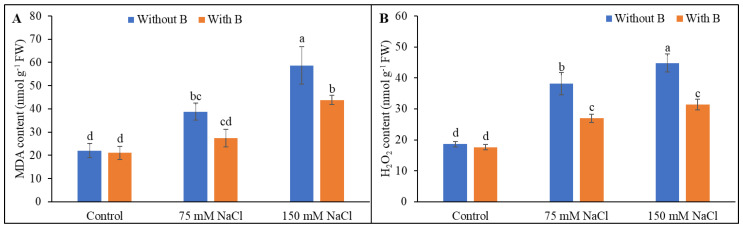
MDA (**A**) and H_2_O_2_ content (**B**) of soybean as affected by salt stress with or without B supplementation. Twenty-day-old plants were imposed with two levels of salt (75 and 150 mM NaCl, 30 days) and a set of plants were supplemented with B (1 mM boric acid). Control treatments were grown without salt. Mean (±SD) was computed from three replicates for each treatment. Different letters over the bars indicate significant differences at *p* ≤ 0.05 applying Tukey’s HSD test.

Malondialdehyde content is a proxy for membrane lipid peroxidation and was increased by salt stress. The H_2_O_2_ levels showed a similar increase in salt-treated soybean seedlings. B addition decreased the MDA level by 38% (under severe salinity stress) and decreased the H_2_O_2_ level by 29% and 30% under mild and severe salt stress, respectively, compared to salt-treated plants without B supplementation (Figure 4).

### 3.4. Ascorbate and Glutathione Pool

**Figure 5:**
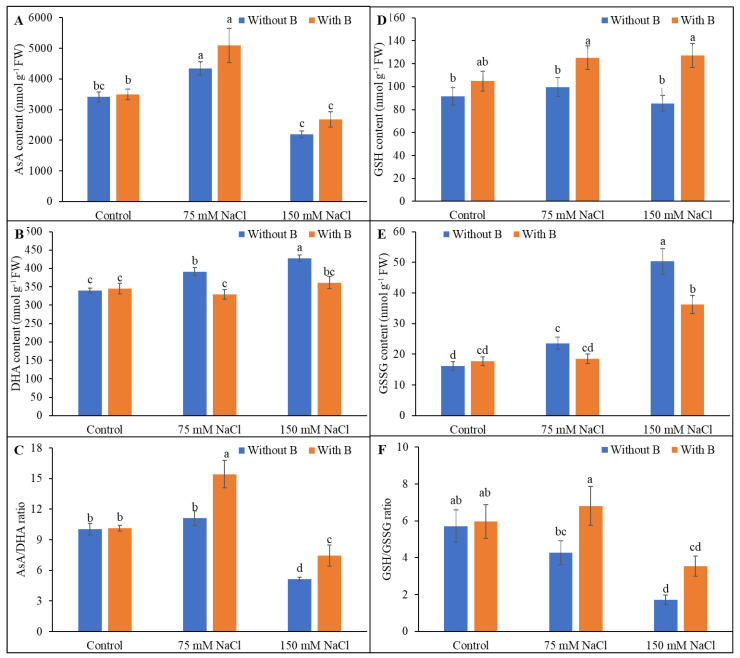
Ascorbate (**A**–**C**) and glutathione (**D**–**F**) pool of soybean as affected by salt stress with or without B supplementation. Twenty-day-old plants were imposed with two levels of salt (75 and 150 mM NaCl, 30 days) and a set of plants were supplemented with B (1 mM boric acid). Control treatments were grown without salt. Mean (±SD) was computed from three replicates for each treatment. Different letters over the bars indicate significant differences at *p* ≤ 0.05 applying Tukey’s HSD test.

The AsA content was increased by exposure to mild salt stress but decreased under severe salt stress. The DHA level increased at both levels of salinity. Together, these changes resulted in a decrease in the AsA/DHA ratio in salt-treated soybean seedlings. However, the AsA/DHA ratio increased after B supplementation by 39% and 45% under mild and severe salt stress, respectively, largely due to the increase in AsA and decrease in DHA levels following B addition (Figure 5A–C).

The glutathione level was unaltered by salt treatment. The GSSG level was greatly increased by salt treatment and increased with the increase in the salinity level. The ratio of GSH/GSSG decreased in salt-treated soybean plants. B supplementation augmented the GSH level under mild and severe salt stress by 26% and 49%, respectively, and diminished GSSG by 22% and 28%, respectively, compared to salt-stressed plants not supplemented with B. These changes explained the maintenance of the GSH/GSSG ratio by B in the salt-treated plants (Figure 5D–F).

### 3.5. Activity of AsA-GSH Pathway Enzymes

**Figure 6:**
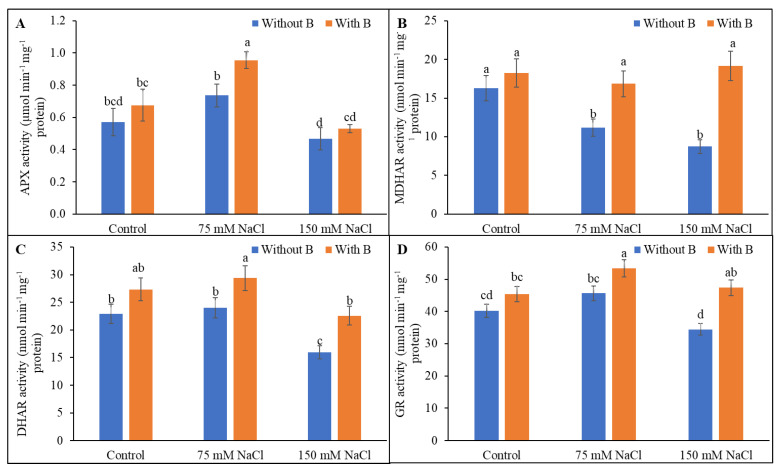
Activities of AsA-GSH pathway enzymes, APX (**A**), MDHR (**B**), DHAR (**C**), and GR (**D**) of soybean as affected by salt stress with or without B supplementation. Twenty-day-old plants were imposed with two levels of salt (75 and 150 mM NaCl, 30 days) and a set of plants were supplemented with B (1 mM boric acid). Control treatments were grown without salt. Mean (±SD) was computed from three replicates for each treatment. Different letters over the bars indicate significant differences at *p* ≤ 0.05 applying Tukey’s HSD test.

The AsA metabolic and recycling enzymes differentially affected under salt stress. APX activity decreased under severe salt stress. The MDHAR and DHAR activity decreased under salt stress compared to unstressed controls. Boron supplementation increased the APX level under mild salinity but not under severe salinity. The MDHAR and DHAR activity was increased by B supplementation of salt-treated plants (Figure 6B,C). Glutathione reductase activity was unchanged by mild salt stress but was increased by severe salt stress compared to unstressed controls. Addition of B to salt-stressed plants increased GR activity compared to salt-stressed plants without B supplementation (Figure 6D).

### 3.6. Methylglyoxal Detoxification System

**Figure 7:**
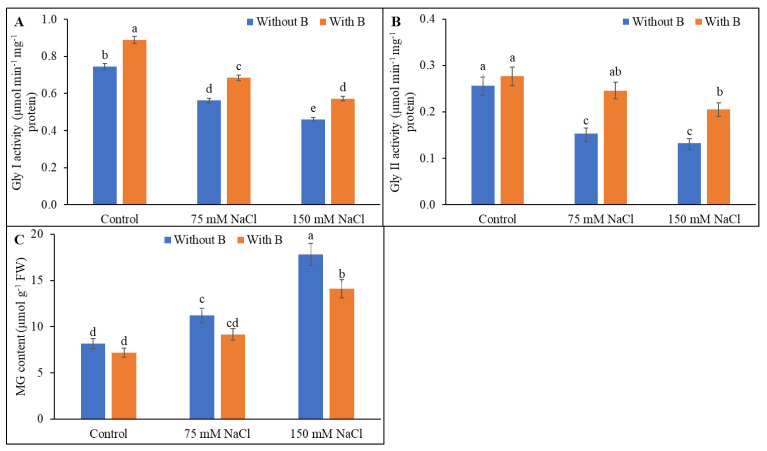
Activities of glyoxalase I (**A**), glyoxalase II (**B**), and level of MG (**C**) of soybean as affected by salt stress with or without B supplementation. Twenty-day-old plants were imposed with two levels of salt (75 and 150 mM NaCl, 30 days) and a set of plants were supplemented with B (1 mM boric acid). Control treatments were grown without salt. Mean (±SD) was computed from three replicates for each treatment. Different letters over the bars indicate significant differences at *p* ≤ 0.05 applying Tukey’s HSD test.

Methylglyoxal levels were increased strongly, by 36% and 118% following exposure to mild and severe salt stress, respectively, compared to unstressed controls. The methylglyoxal level was decreased by 21% in severely stressed plants following B supplementation. Glyoxalase I and Gly II activities were decreased by mild salt stress and further decreased by severe salt stress. Glyoxalase I and Gly II activities were increased in salt-stressed plants supplemented with B compared to salt-stressed plants without B supplementation (Figure 7).

## 4. Discussion

Growth inhibition is the most common deleterious effect of salt exposure. The aim of the present study was to determine the various factors that contribute to this inhibition. Plants growing in saline conditions experience a reduction in the rate of water absorption due to altered osmotic potential. This is accompanied by a lower uptake of essential mineral nutrients, in part due to competition with Na [4]. Poor root growth under salt stress further hinders water and nutrient uptake, as well as transport. Photosynthesis is also hindered under salinity [5]. These alterations are the common reasons for salt-induced growth reduction [4,31]. 

Supplementation with B increased the shoot fresh weight of salt-treated plants. Several other studies have also demonstrated a B-induced improvement in growth parameters of plants growing under saline conditions [3,16]. However, the differences observed in the growth parameters of salt-stressed soybean plants in response to B application were not statistically significant in the present study. Further studies with different doses of B are needed to confirm the potential for a growth-enhancing role of B under salt stress in soybean. 

Salt stress imposes oxidative stress that causes Chl pigment breakdown. Chl biosynthesis is also hampered during salt exposure due to nutrient imbalance and metabolic disruptions [3,4,11]. In the present study, salt stress reduced the Chl content in the soybean plants, but B supplementation did not mitigate the Chl losses significantly. Boron plays a role in cytoskeletal protein construction and nitrogen metabolism, which are both required for Chl synthesis [12,13,14]. Restoration of Chl levels has been reported in salt-stressed rose [16] and potato [3] plants following B application. Therefore, restoration of the Chl content in soybean may depend on the dosage, duration, or application method. Future studies should examine these possibilities to determine if better results can be achieved.

Pro accumulation and reduced RWC indicate the induction of osmotic stress in soybean plants under salt stress. A higher accumulation of Pro and other osmolytes is desirable to combat the osmotic stress induced by any environmental stress [4]. Increased Pro biosynthesis and accumulation have been reported in response to B in previous studies, which also showed improvements in plant water status [3,32]. Boron is involved in nitrogen metabolism and in the synthesis of some of the secondary metabolites connected to the biosynthesis of osmoprotectants such as Pro [14]. In the present study, B supplementation of salt-treated plants increased RWC but had no effect on Pro levels. This finding might reflect that plants have a number of endogenous osmoprotectant molecules that can regulate the water content of plants under stress conditions. Boron might, therefore, have a role in regulating the levels of osmoprotectants other than Pro and/or in regulating other physiological attributes that can improve the RWC of salt-stressed plants. Further investigation is needed to better understand the effects of B on osmoregulation.

Salinity causes osmotic stress that, in turn, creates oxidative stress and inhibits biomembrane functions. Ion toxicity also causes oxidative damage to biomembranes. Inhibition of antioxidant enzyme activity under salt stress increases ROS production and causes oxidative damage [10,33]. Soybean plants exposed to salinity showed increased generation of H_2_O_2_, which then damaged the membranes, as indicated by increased MDA levels. Electrolyte leakage was also higher during salt exposure, further confirming membrane damage. These common responses to oxidative stress have been documented in many different plant species during salt exposure [4,8,34]. In the present study, exogenous B supplementation decreased the salinity-induced oxidative stress, as confirmed by the decreased levels of H_2_O_2_, MDA, and electrolyte leakage from the salt-treated soybean plants. 

Previous studies of B effects on salt stress responses have verified a role for B in oxidative stress mitigation. Decreases in lipoxygenase activity, electrolyte leakage, and MDA and H_2_O_2_ levels by B were reported in salt-stressed *Pistacia vera* leaves [7]. The MDA and H_2_O_2_ contents were diminished by B application in aluminum-stressed plants [35,36]. Downregulation of H_2_O_2_ production, as well as membrane lipid peroxidation, by B supplementation was confirmed in citrus plants under aluminum toxicity stress [9].

Salt stress generates a series of damaging secondary effects, including altered biosynthesis of metabolites and inhibition of enzyme activities [3,10,11,33]. In the present study, salt stress altered AsA and DHA levels, thereby decreasing the AsA/DHA ratio, whereas B supplementation reversed the damaging effects and restored the AsA/DHA ratio. Ascorbate peroxidase activity was decreased by severe salinity (but was unaltered under mild salt stress), and MDHAR and DHAR activities were decreased in salt-treated soybean plants, and these decreases were correlated with the decreases in AsA levels and the AsA/DHA ratio. Boron supplementation of the salt-treated soybean plants increased the MDHAR and DHAR activity and helped to restore the AsA level and decrease the DHA level, thereby increasing the AsA/DHA ratio above that observed in salt-stressed plants.

The GSH level was not altered in salt-treated soybean plants, but the GSSG level was greatly increased, resulting in a strongly decreased GSH/GSSG ratio under saline conditions compared to unstressed plants. B supplementation of the salt-stressed plants raised the GSH level and decreased the GSSG level, thereby increasing the GSH/GSSG ratio above that of salt-stressed plants. Glutathione reductase activity in salt-treated plants was increased by B supplementation, providing further improvement in the GSG/GSSG ratio. 

Research that focuses on a potential regulatory role for B in antioxidant defense systems is sparse. The application of Bio-B fertilizer was shown to ameliorate freezing injury in grapevine through augmentation of activities of antioxidant enzymes, such as CAT, peroxidase (POD), and SOD [35]. The activity of APX and CAT and the levels of nonenzymatic antioxidant compounds, such as AsA and phenolic compounds, were enhanced considerably in *Pistacia vera* by B addition, and the trees showed better salinity tolerance following B supplementation [7]. Citrus plants under aluminum toxicity showed responses to B supplementation that included upregulated SOD activity and reduced activities of peroxidase, CAT, and polyphenol oxidase, as well as lower protein and proline levels [9]. Riaz et al. [15,36] confirmed that B supply decreased aluminum toxicity responses in trifoliate orange and increased the levels of antioxidant system components, including the activities of peroxidase, CAT, APX, phenylalanine ammonia lyase, and polyphenol oxidase, and the accumulation of Pro and secondary metabolites, while also improving components of the cell wall [36].

MG is produced through various abiotic stresses and has toxic effects on cell metabolism. Scavenging of MG through the glyoxalase system is a prerequisite for improving salt tolerance [4,8]. Gly I and Gly II activities were diminished and MG levels were increased in salt-treated soybean plants, and B application reversed these effects, leading to decreased MG accumulation. An increase in glyoxalase enzyme activity was also shown to decrease the MG content in salt-affected mung bean [4] and tomato (Parvin et al., 2020), in agreement with the present findings. However, a role for B in the maintenance of the glyoxalase system still requires investigation.

## 5. Conclusions

The present study revealed that B supplementation improved water relations of the salt-affected plants. Exogenous B also enhanced many components of the plant antioxidant defense system, including GSH, GSH/GSSG, AsA/DHA, and activities of APX, MDHAR, DHAR, and GR, to mitigate oxidative damage. Overall, B supplementation decreased the MDA level and electrolyte leakage in salt-treated soybean plants, while stimulating the methylglyoxal detoxification process through upregulation of the components of the glyoxalase system, including GSH level and Gly I and Gly II activities. B is a recognized fertilizer micronutrient with vital physiological and developmental functions [12,13,14], but few studies have probed the protective role of B under different abiotic stresses [3,9,16,36]. Therefore, many aspects remain to be revealed regarding the protective role of B in plants exposed to environmental stress. Combined application of B with other trace elements and their physiological roles also warrants further research. 

## Data Availability

All data are available in this manuscript.
